# Supplementary material for: Molecular Mapping of Urinary Complement Peptides in Kidney Diseases
Source: Proteomes. 2021 Dec 13;9(4):49. doi: 10.3390/proteomes9040049 (PMC8709096; doi:10.3390/proteomes9040049)

e06787

Sequence: IGGLRNNNEKDMALT, Charge: +2, Monoisotopic m/z: 823.41852 Da (-1.44 mmu/-1.74 ppm), MH+: 1645.82976 Da, RT: 60.2435 min,
Identified with: Sequest HT (v1.17); XCorr:2.81,
Fragment match tolerance used for search: 0.05 Da
Fragments used for search: $-H_2O$; y; $-NH_3$; y; b; b; $-H_2O$; b; $-NH_3$; y

Protein:
- Complement C3

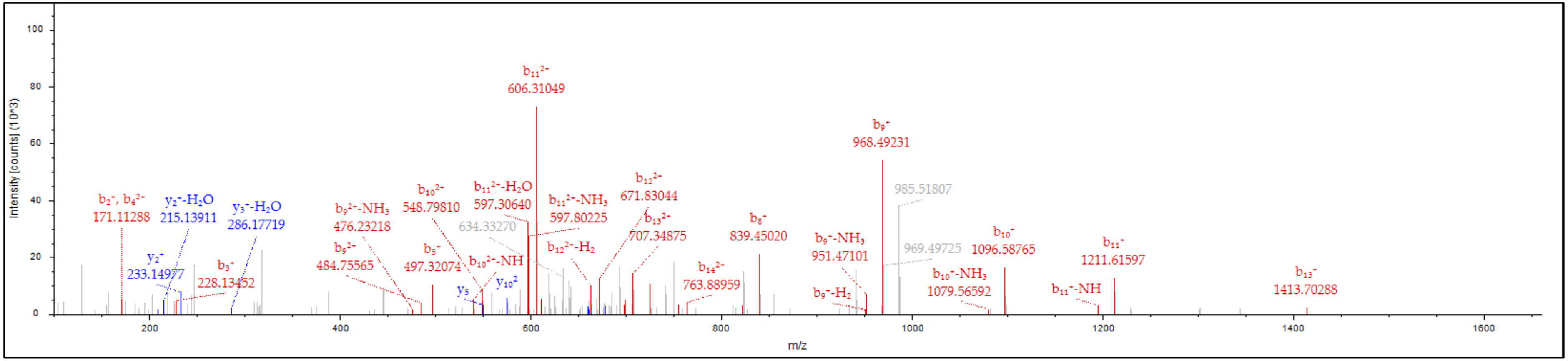

e08849

Sequence: YQKDAPDHQELNLDVS, Charge: +2, Monoisotopic m/z: 936.44110 Da (-1.04 mmu/-1.11 ppm), MH+: 1871.87493 Da,
Identified with: Sequest HT (v1.17); XCorr:2.58,
Fragment match tolerance used for search: 0.05 Da
Fragments used for search: $-H_2O$; y; $-NH_3$; y; b; b; $-H_2O$; b; $-NH_3$; y

Protein:
- Complement C3

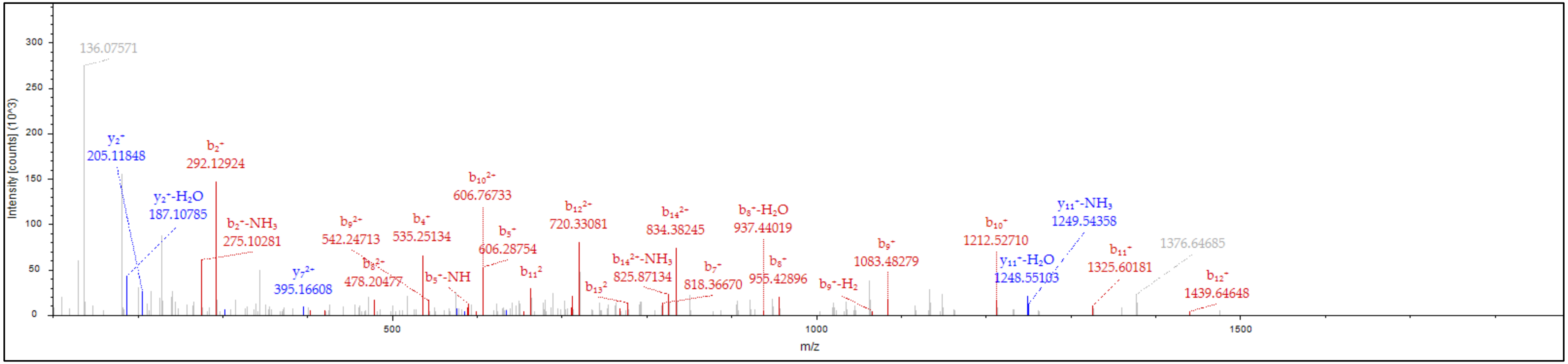

<u>e09686</u>

Sequence: QALAQYQKDAPDHQELN, Charge: +3, Monoisotopic m/z: 656.98444 Da (-0.75 mmu/-1.14 ppm), MH+: 1968.93875 Da,
Identified with: Sequest HT (v1.17); XCorr:4.46,
Fragment match tolerance used for search: 0.05 Da
Fragments used for search: $-H_2O$; y; $-NH_3$; y; b; b; $-H_2O$; b; $-NH_3$; y

Protein:
- Complement C3

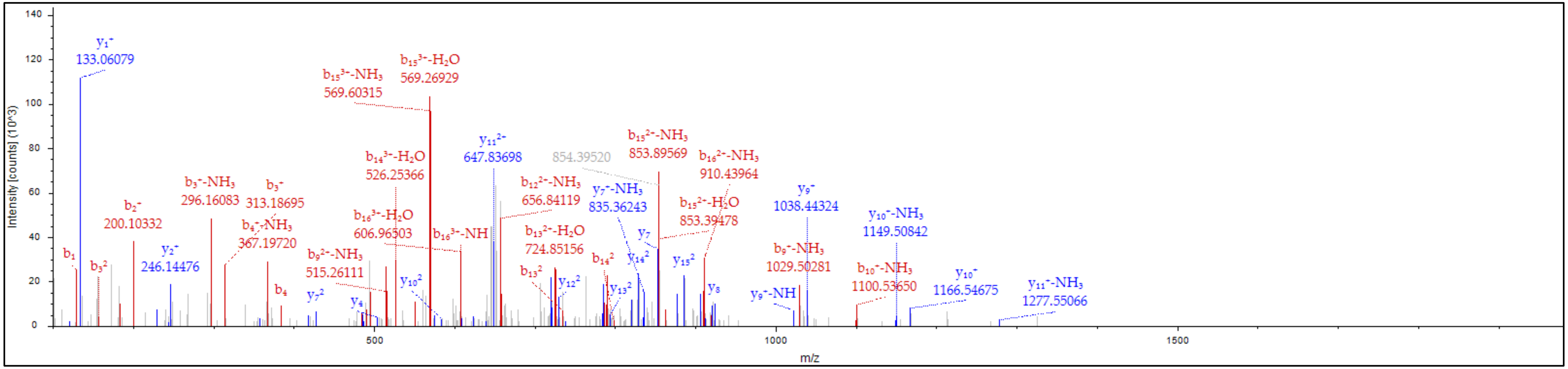

e14381

Sequence: LTTAKDKNRWEDPGKQLYNVEAT, Charge: +3, Monoisotopic m/z: 893.12323 Da (-0.96 mmu/-1.08 ppm), MH+: 2677.35514 Da,
Identified with: Sequest HT (v1.17); XCorr:4.74,
Fragment match tolerance used for search: 0.05 Da
Fragments used for search: -H_2O; y; -NH_3; y; b; b; -H_2O; b; -NH_3; y

Protein:
- Complement C3

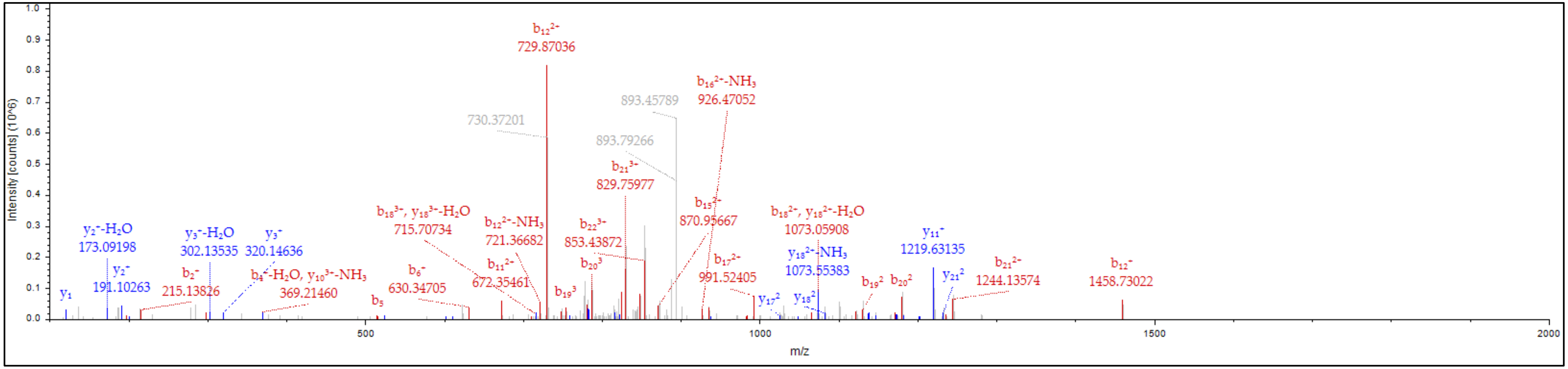

e16109

Sequence: LTTAKDKNRWEDPGKQLYNVEATSYA, Charge: +3,   Monoisotopic m/z: 1000.16779 Da (-0.56 mmu/-0.56 ppm),   MH+: 2998.48880 Da,
Identified with: Sequest HT (v1.17); XCorr:4.02,
Fragment match tolerance used for search: 0.05 Da
Fragments used for search: $-H_2O$; y; $-NH_3$; y; b; b; $-H_2O$; b; $-NH_3$; y

Protein:
- Complement C3

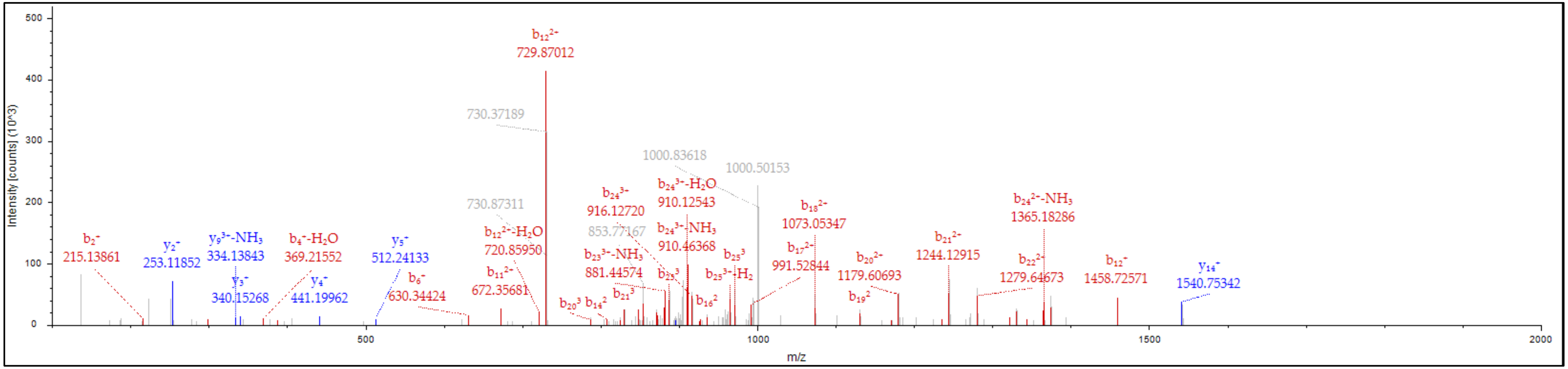

e08566

Sequence: TETIEGVDAEDGHGPGEQ, Charge: +2, Monoisotopic m/z: 920.89429 Da (-0.93 mmu/-1.01 ppm), MH+: 1840.78130 Da,
Identified with: Sequest HT (v1.17); XCorr:4.35,
Fragment match tolerance used for search: 0.05 Da
Fragments used for search: -H_2O; y; -NH_3; y; b; b; -H_2O; y

Protein:
- Complement factor B

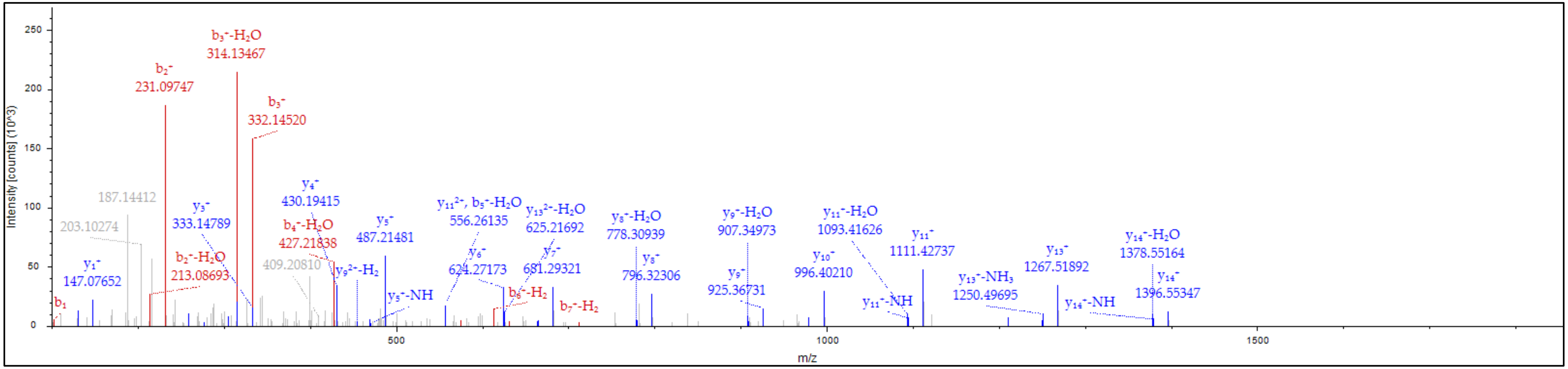

e11594

Sequence: LSSLTETIEGVDAEDGHGPGEQ, Charge: +2, Monoisotopic m/z: 1121.00940 Da (-1.91 mmu/-1.7 ppm), MH+: 2241.01152 Da,
Identified with: Sequest HT (v1.17); XCorr:2.36,
Fragment match tolerance used for search: 0.05 Da
Fragments used for search: -H_2O; y; -NH_3; y; b; b; -H_2O; y

Protein:
- Complement factor B

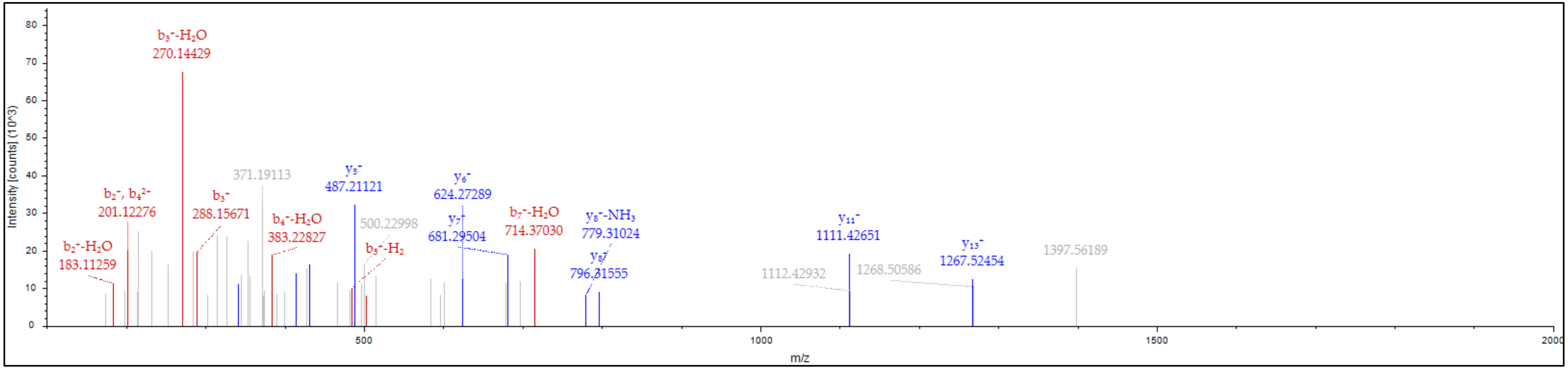

e09265

Sequence: DELPAKDDPDAPLQPVTP, Charge: +2, Monoisotopic m/z: 959.47534 Da (-0.3 mmu/-0.32 ppm), MH+: 1917.94341 Da,
Identified with: Sequest HT (v1.17); XCorr:3.22,
Fragment match tolerance used for search: 0.05 Da
Fragments used for search: $-H_2O$; y; $-NH_3$; y; b; b; $-H_2O$; b; $-NH_3$; y

Protein:
- Complement C4-B

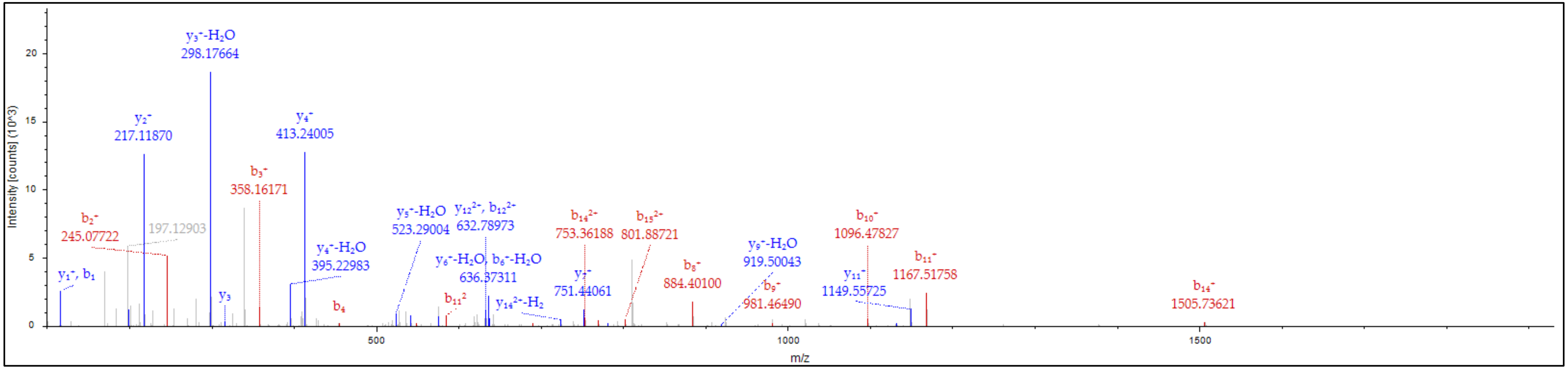

e08161

Sequence: TLTKAPADLRGVAHNNL, Charge: +3,   Monoisotopic m/z: 597.66779 Da (+0.55 mmu/+0.92 ppm),   MH+: 1790.98880 Da,
Identified with: Sequest HT (v1.17); XCorr:2.95,
Fragment match tolerance used for search: 0.05 Da
Fragments used for search: $-H_2O$; y; $-NH_3$; y; b; b; $-H_2O$; b; $-NH_3$; y

Protein:
- Complement C4-B

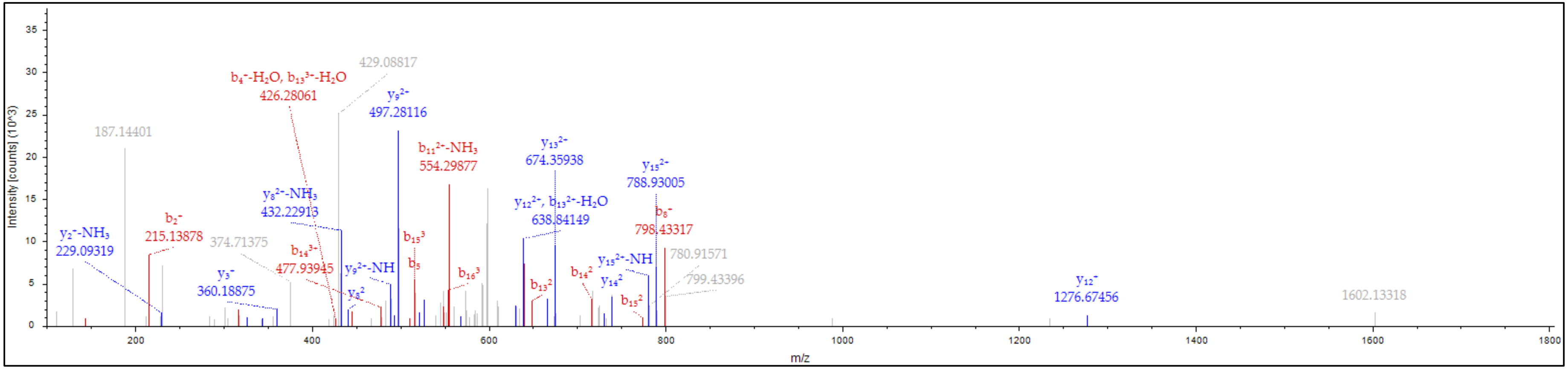

e13403

Sequence: FLSSLTETIEGVDAEDGHGPGEQQ, Charge: +2,   Monoisotopic m/z: 1258.57336 Da (-1.44 mmu/-1.14 ppm),   MH+: 2516.13945 Da,
Identified with: Sequest HT (v1.17); XCorr:2.03,
Fragment match tolerance used for search: 0.05 Da
Fragments used for search: $-H_2O$; y; $-NH_3$; y; b; b; $-H_2O$; b; $-NH_3$; y

Protein:
- Complement factor B

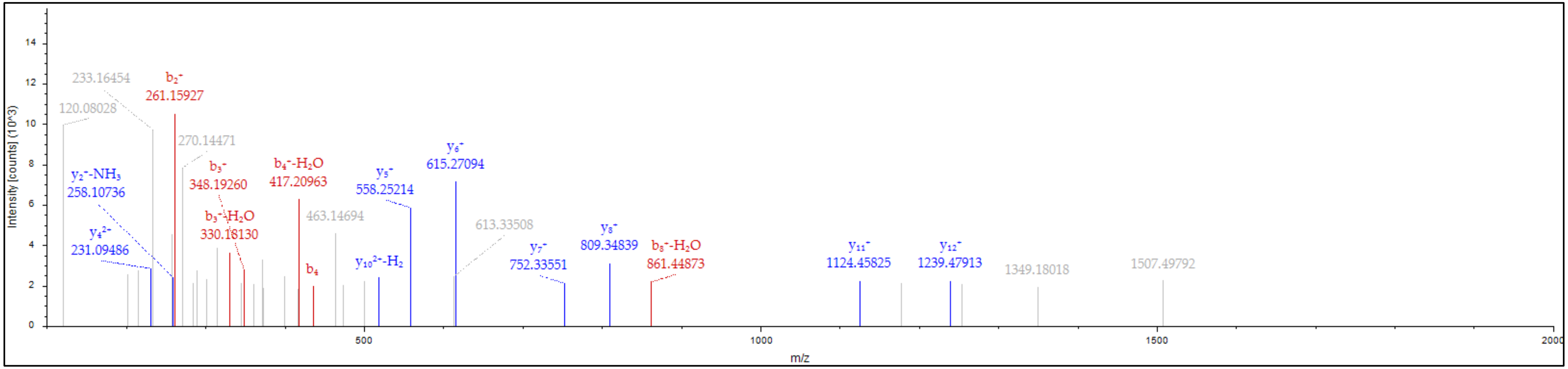

e00117

Sequence: LGVAHNNL, Charge: +2,   Monoisotopic m/z: 419.23227 Da (-0.23 mmu/-0.56 ppm),   MH+: 837.45726 Da,   RT: 53.4069 min,
Identified with: Sequest HT (v1.17); XCorr:2.11,
Fragment match tolerance used for search: 0.05 Da
Fragments used for search: $-NH_3$; y; b; b; $-NH_3$; y

Protein:
- Complement C4-A

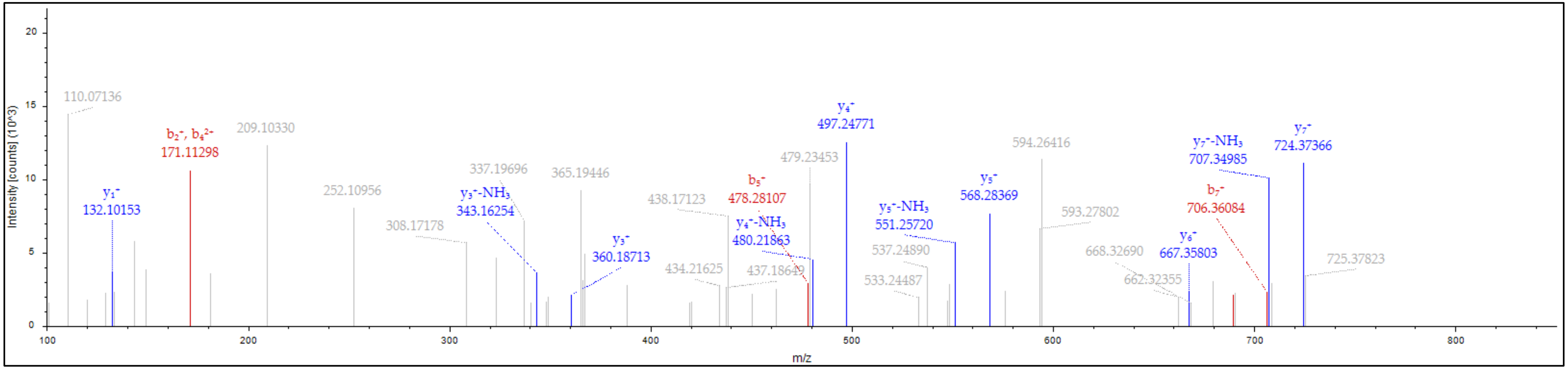

e11703

Sequence: SSLTETIEGVDAEDGHGPGEQQ, Charge: +2, Monoisotopic m/z: 1128.49744 Da (-1.13 mmu/-1 ppm), MH+: 2255.98760 Da,
Identified with: Sequest HT (v1.17); XCorr:2.66,
Fragment match tolerance used for search: 0.05 Da
Fragments used for search: $-H_2O$; y; $-NH_3$; y; b; b; $-H_2O$; b; $-NH_3$; y

Protein:
- Complement factor B

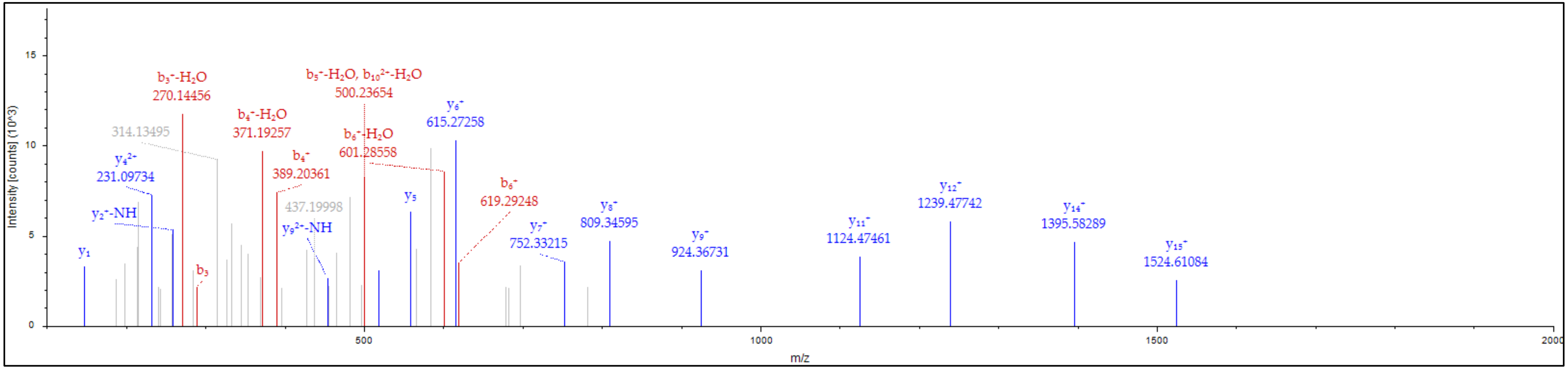

e12606

Sequence: LSSLTETIEGVDAEDGHGPGEQQ, Charge: +3, Monoisotopic m/z: 790.36224 Da (-0.58 mmu/-0.73 ppm), MH+: 2369.07218 Da,
Identified with: Sequest HT (v1.17); XCorr:4.38,
Fragment match tolerance used for search: 0.05 Da
Fragments used for search: $-H_2O$; y; $-NH_3$; y; b; b; $-H_2O$; b; $-NH_3$; y

Protein:
- Complement factor B

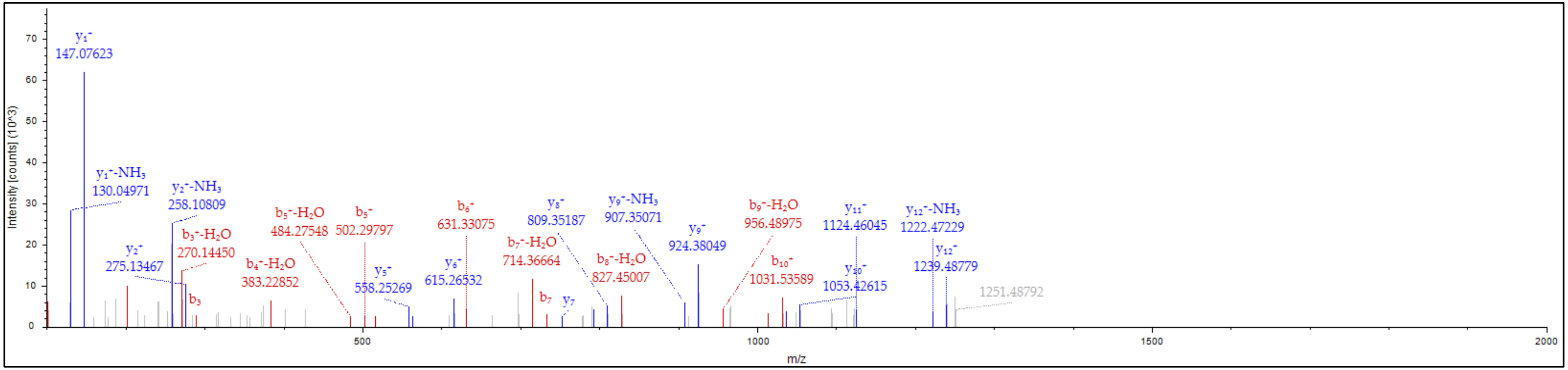

e09429

Sequence: EGVQKEDIPPADLSDQVP, Charge: +2,   Monoisotopic m/z: 968.97766 Da (-0.89 mmu/-0.92 ppm),   MH+: 1936.94805 Da,
Identified with: Sequest HT (v1.17); XCorr:2.81,
Fragment match tolerance used for search: 0.05 Da
Fragments used for search: -H_2O; y; -NH_3; y; b; b; -H_2O; b; -NH_3; y

Protein:
- Complement C3

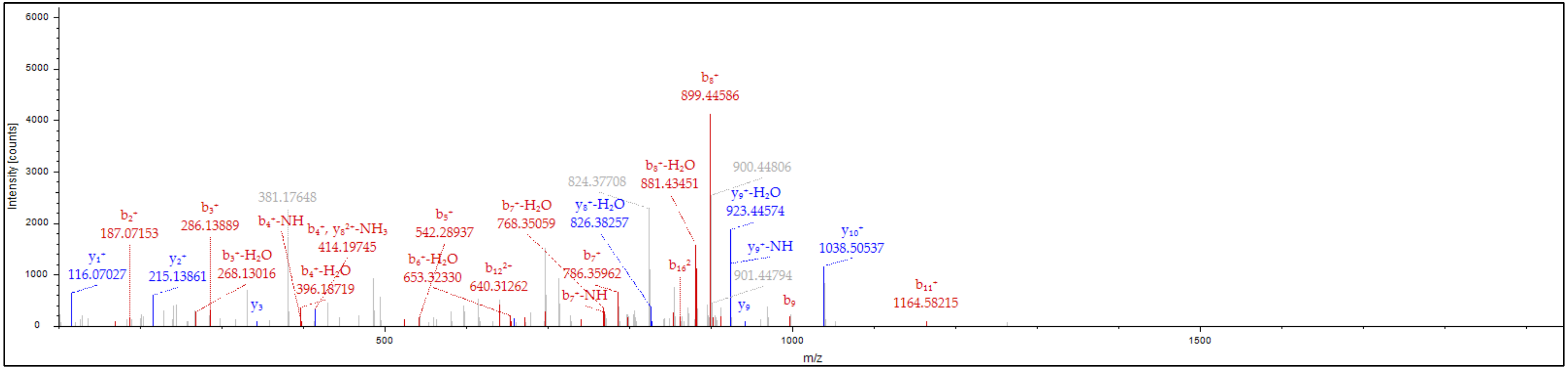

e17084

Sequence: EGVQKEDIPPADLSDQVPDTESETRILLQ, Charge: +3, Monoisotopic m/z: 1074.87073 Da (-1.2 mmu/-1.11 ppm), MH+: 3222.59763 Da,
Identified with: Sequest HT (v1.17); XCorr:3.40,
Fragment match tolerance used for search: 0.05 Da
Fragments used for search: -H_2O; y; -NH_3; y; b; b; -H_2O; b; -NH_3; y

Protein:
- Complement C3

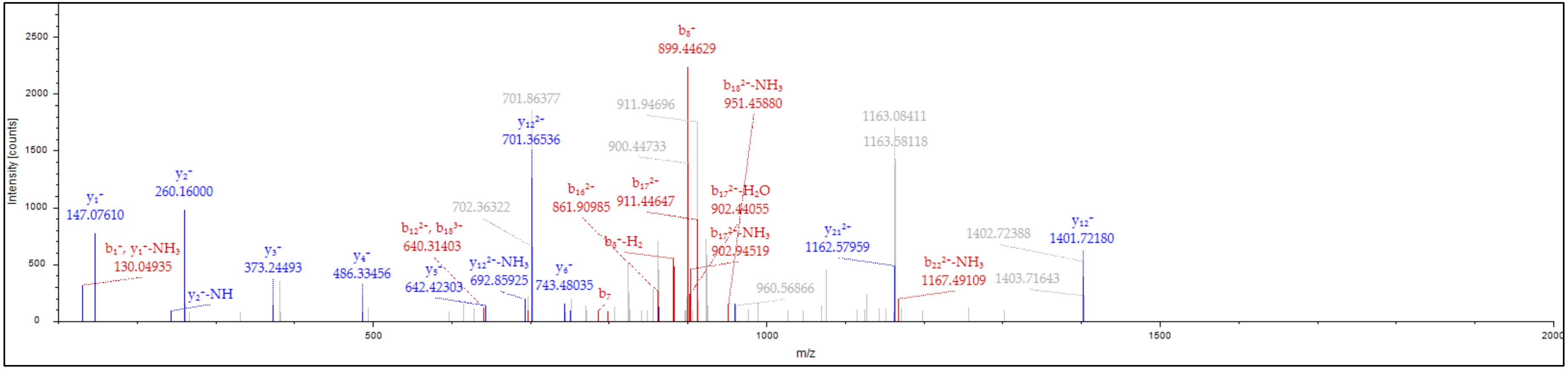

e18666

Sequence: EGVQKEDIPPADLSDQVPDTESETRILLQGTPVA, Charge: +3, Monoisotopic m/z: 1216.61108 Da (-3.32 mmu/-2.73 ppm), MH+: 3647.81870 Da,
Identified with: Sequest HT (v1.17); XCorr:2.23,
Fragment match tolerance used for search: 0.05 Da
Fragments used for search: -H_2O; y; -NH_3; y; b; b; -H_2O; b; -NH_3; y

Protein:
- Complement C3

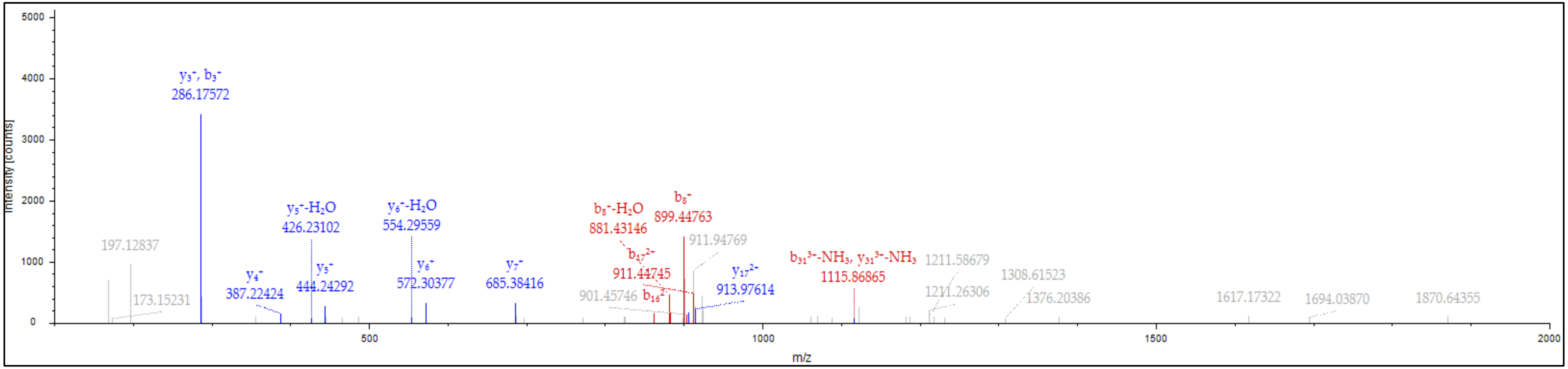

e12939

Sequence: LQGTPVAQMTEDAVDAERLKHL, Charge: +4, Monoisotopic m/z: 606.31476 Da (-0.57 mmu/-0.94 ppm), MH+: 2422.23720 Da,
Identified with: Sequest HT (v1.17); XCorr:2.93,
Fragment match tolerance used for search: 0.05 Da
Fragments used for search: -H_2O; y; -NH_3; y; b; b; -H_2O; b; -NH_3; y

Protein:
- Complement C3

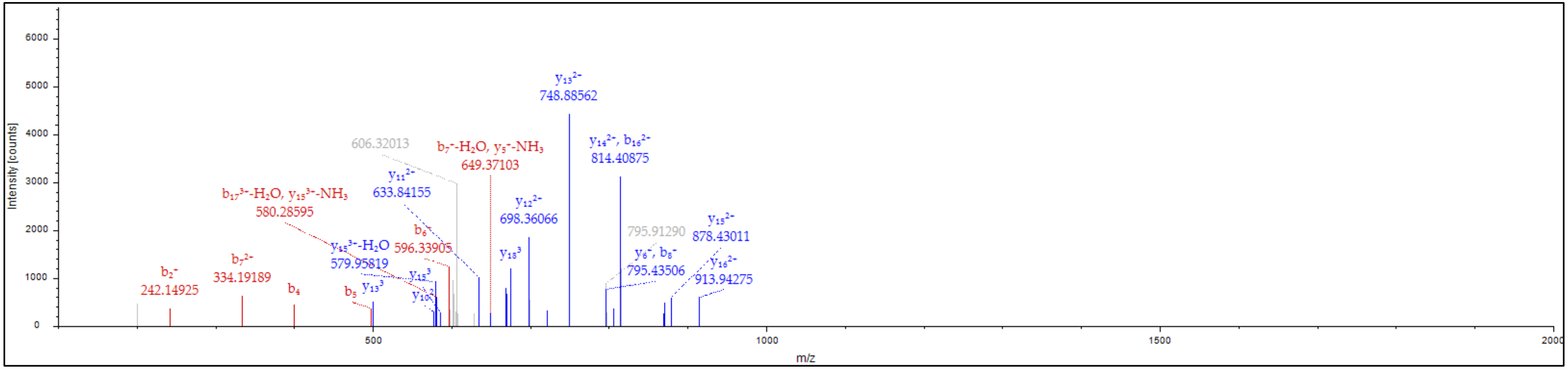

e06142
Sequence: TKAPADLRGVAHNNL, Charge: +3, Monoisotopic m/z: 526.28931 Da (-0.68 mmu/-1.3 ppm), MH+: 1576.85337 Da,
Identified with: Sequest HT (v1.17); XCorr:3.67,
Fragment match tolerance used for search: 0.05 Da
Fragments used for search: $-H_2O$; y; $-NH_3$; y; b; b; $-H_2O$; b; $-NH_3$; y

Protein:
- Complement C4-B

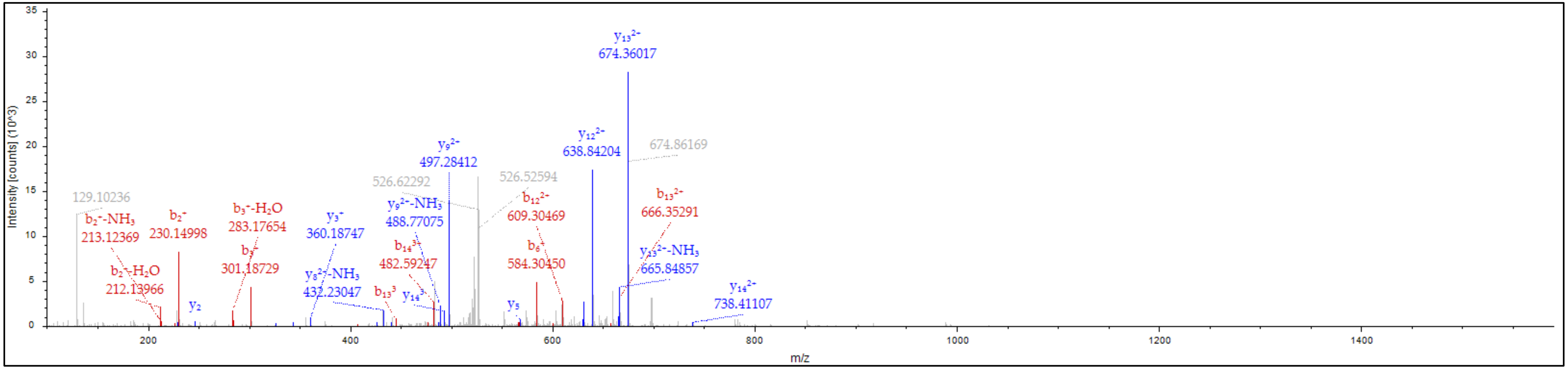

e09682

Sequence: TETIEGVDAEDGHGPGEQQ, Charge: +2, Monoisotopic m/z: 984.92352 Da (-0.98 mmu/-1 ppm), MH+: 1968.83977 Da,
Identified with: Sequest HT (v1.17); XCorr:5.18,
Fragment match tolerance used for search: 0.05 Da
Fragments used for search: $-H_2O$; y; $-NH_3$; y; b; b; $-H_2O$; b; $-NH_3$; y

Protein:
- Complement factor B

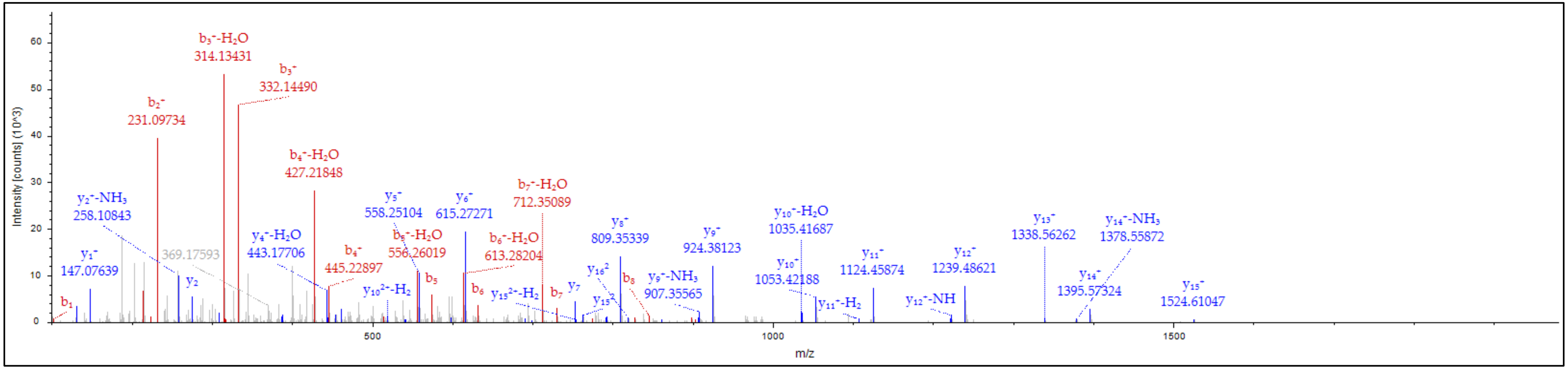

e10517

Sequence: LTETIEGVDAEDGHGPGEQQ, Charge: +2,   Monoisotopic m/z: 1041.46448 Da (-2.06 mmu/-1.98 ppm),   MH+: 2081.92168 Da,
Identified with: Sequest HT (v1.17); XCorr:4.60,
Fragment match tolerance used for search: 0.05 Da
Fragments used for search: $-H_2O$; y; $-NH_3$; y; b; b; $-H_2O$; b; $-NH_3$; y

Protein:
- Complement factor B

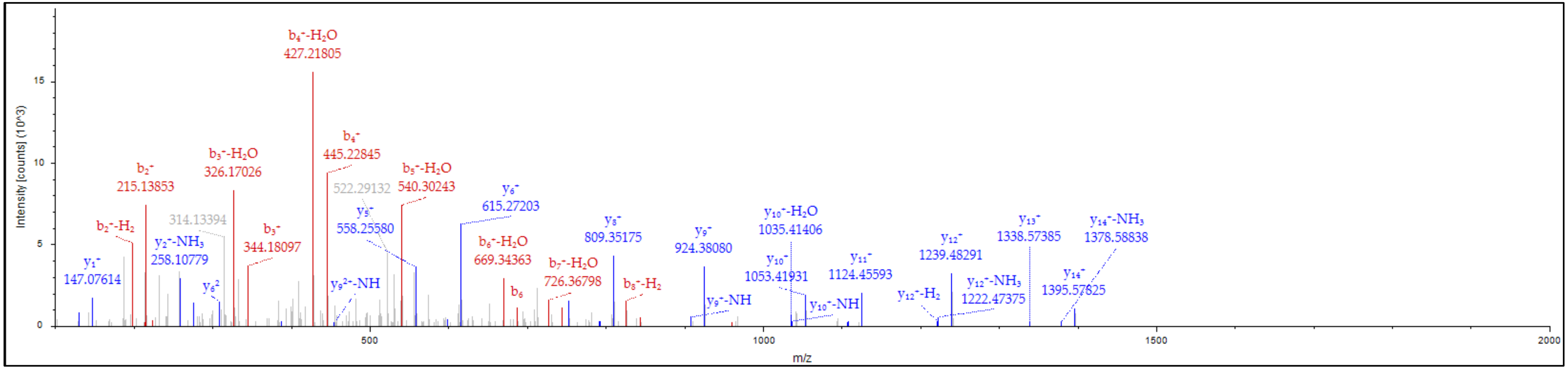

e08035

Sequence: TLTKAPVDLLGVAHNNL, Charge: +3, Monoisotopic m/z: 592.66925 Da (-2.74 mmu/-4.62 ppm), MH+: 1775.99320 Da,
Identified with: Sequest HT (v1.17); XCorr:2.75,
Fragment match tolerance used for search: 0.05 Da
Fragments used for search: $-H_2O$; y; $-NH_3$; y; b; b; $-H_2O$; b; $-NH_3$; y

Protein:
- Complement C4-A

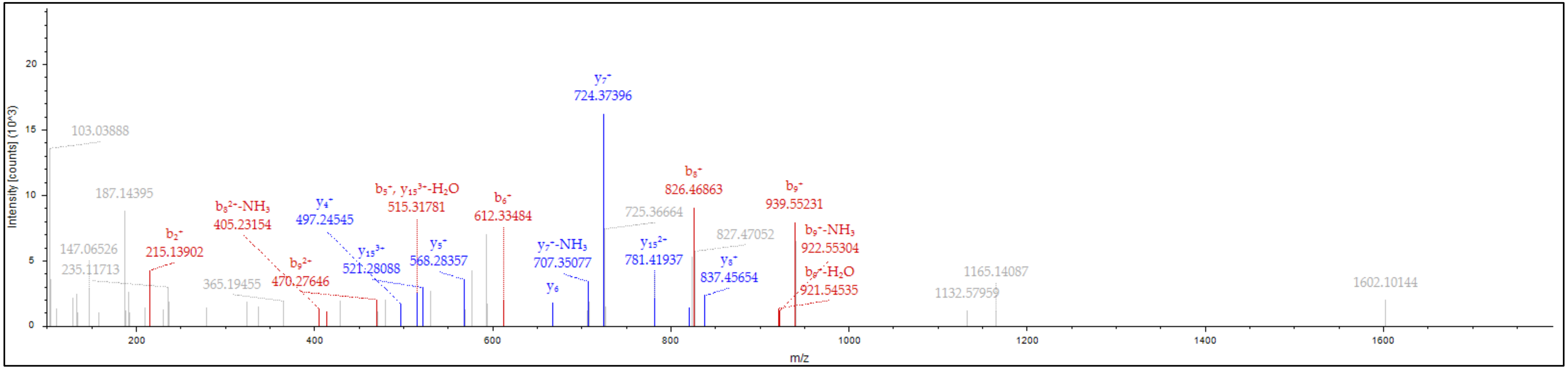

e16041

Sequence: EGVQKEDIPPADLSDQVPDTESETRIL, Charge: +3, Monoisotopic m/z: 994.49005 Da (-0.99 mmu/-1 ppm), MH+: 2981.45560 Da,
Identified with: Sequest HT (v1.17); XCorr:3.55,
Fragment match tolerance used for search: 0.05 Da
Fragments used for search: $-H_2O$; y; $-NH_3$; y; b; b; $-H_2O$; b; $-NH_3$; y

Protein:
- Complement C3

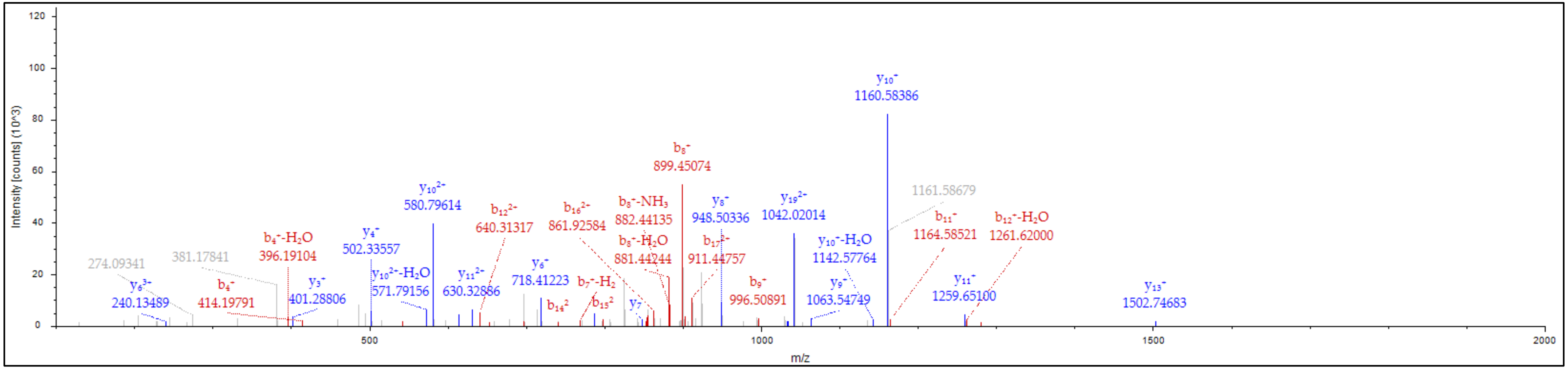

e03984

Sequence: APVDLLGVAHNNL, Charge: +2,   Monoisotopic m/z: 666.86676 Da (-0.39 mmu/-0.59 ppm),   MH+: 1332.72624 Da,
Identified with: Sequest HT (v1.17); XCorr:2.37,
Fragment match tolerance used for search: 0.05 Da
Fragments used for search: $-H_2O$; y; $-NH_3$; y; b; b; $-H_2O$; b; $-NH_3$; y

Protein:
- Complement C4-A

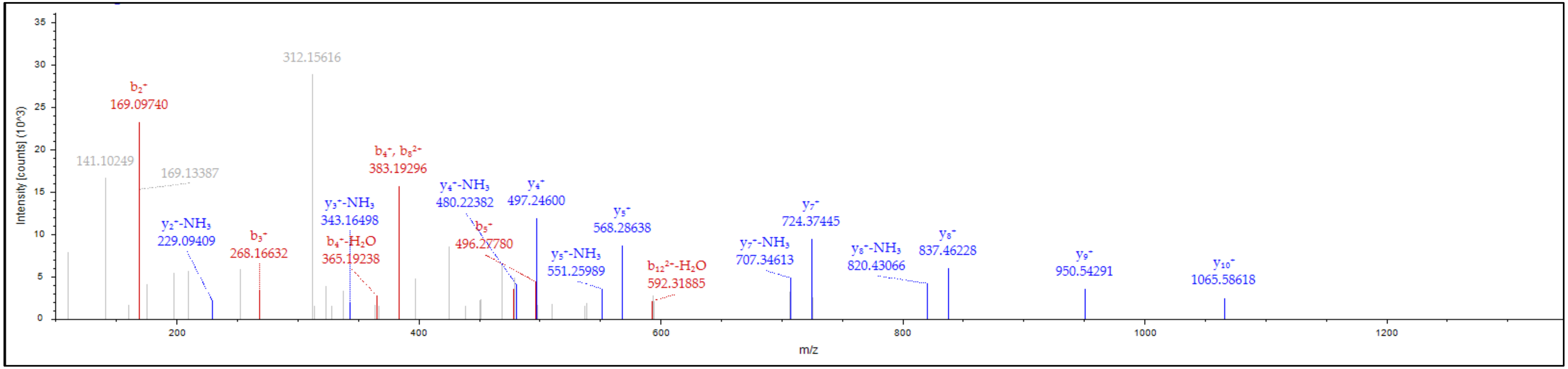

Supplement: Supplementary file 1 [file proteomes-09-00049-s001.zip › Figure S1 Tandem mass spectra and assignment of sequences of the 23 complement-derived urine peptides detected.pdf]
