# Supplementary figures and images for: Molecular Mapping of Urinary Complement Peptides in Kidney Diseases
Source: Proteomes. 2021 Dec 13;9(4):49. doi: 10.3390/proteomes9040049 (PMC8709096; doi:10.3390/proteomes9040049)

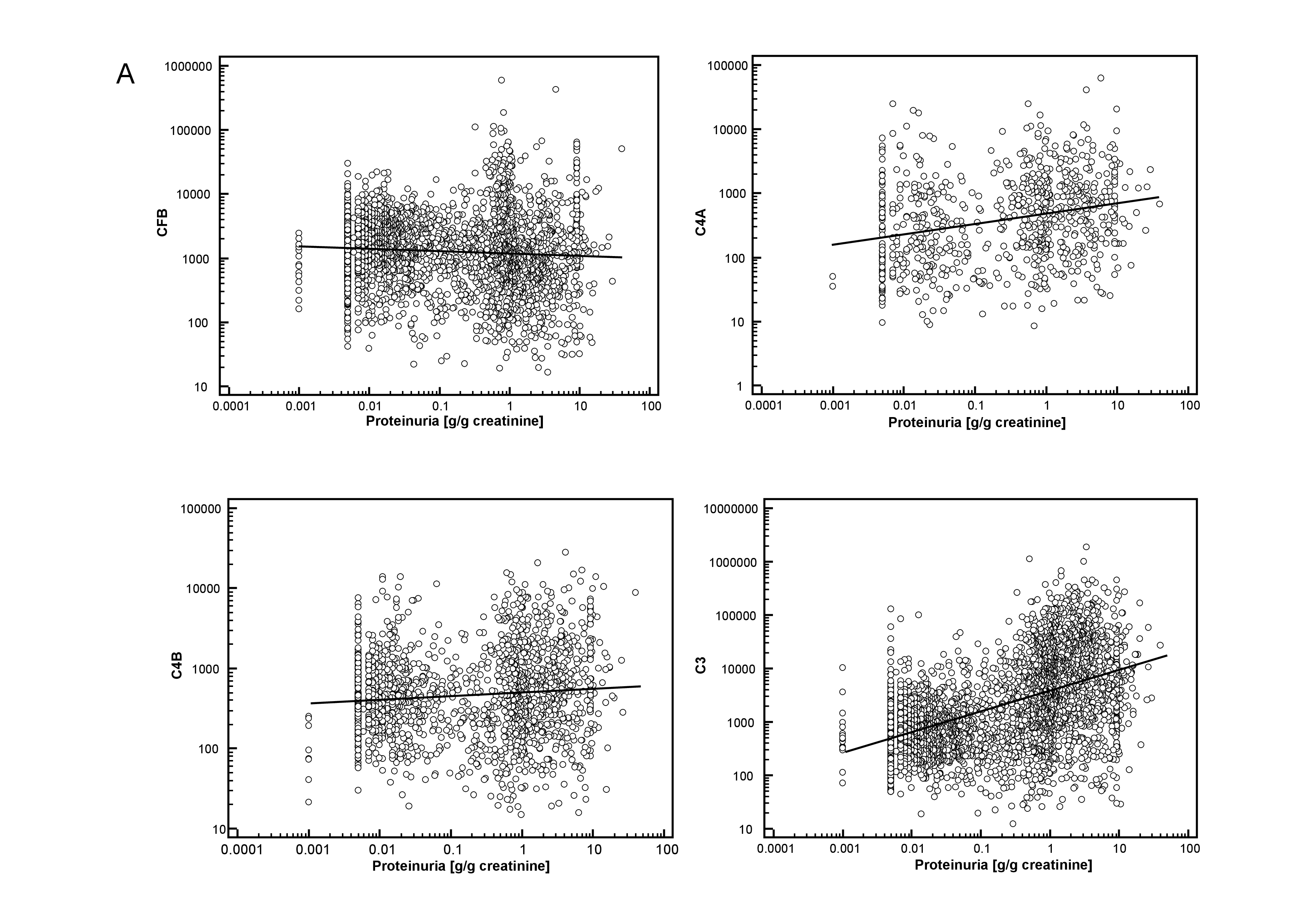

Supplement: Supplementary file 1 [file proteomes-09-00049-s001.zip › Figure S2A Association of the combined abundance of complement peptides (from Complement factor B, 4A, 4B and 3) with proteinuria, prior adjustment for proteinuria.tiff]

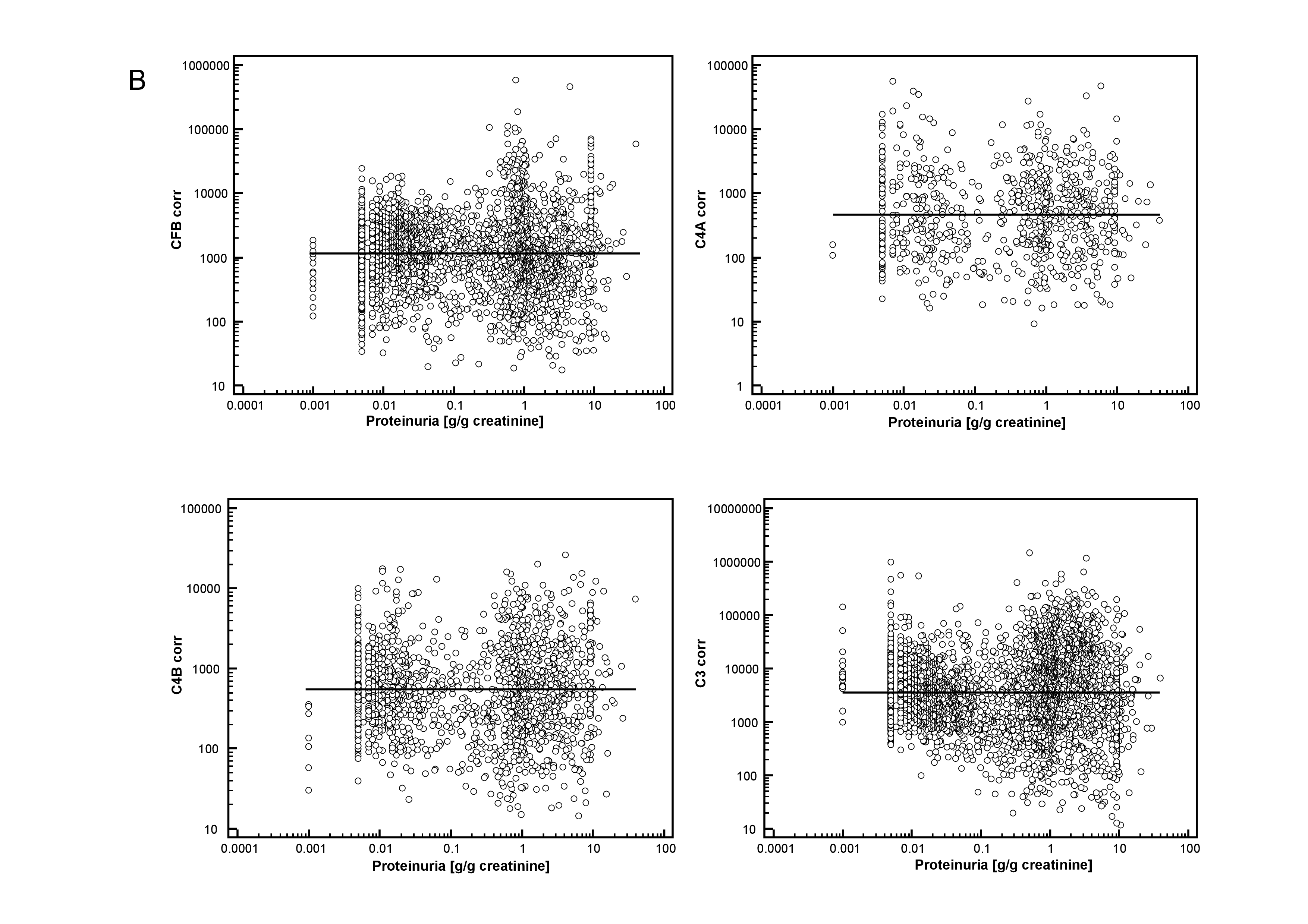

Supplement: Supplementary file 1 [file proteomes-09-00049-s001.zip › Figure S2B Association of the combined abundance of complement peptides (from Complement factor B, 4A, 4B and 3) with proteinuria after adjustment for proteinuria.tiff]

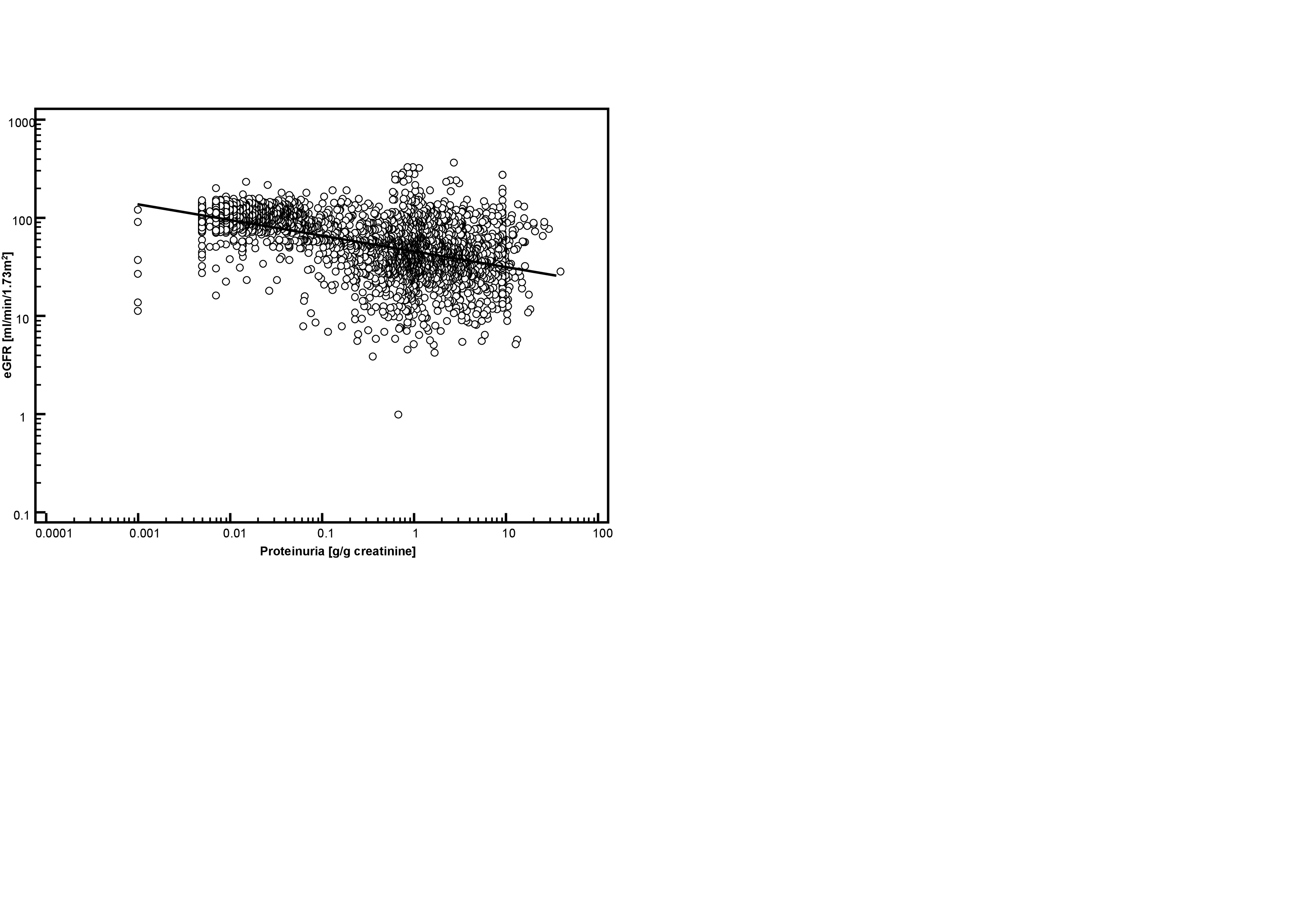

Supplement: Supplementary file 1 [file proteomes-09-00049-s001.zip › Figure S3 Association of the combined abundance of complement peptides (from Complement factor B, 4A, 4B and 3) with proteinuria.tiff]
